# Myoconjunctival Enucleation Study (MES): Outcome of Myoconjunctival Enucleation Technique with Polymethyl Methacrylate (PMMA) implant and Custom-made prosthesis (CMP) in a tertiary eye care center in Nepal

**DOI:** 10.1371/journal.pone.0321703

**Published:** 2025-07-29

**Authors:** Hom Bahadur Gurung, Purnima Rajkarnikar Sthapit, Malita Amatya, Dikshya Bista, Sushant Adiga, Manish Poudel, Raba Thapa, Rohit Saiju

**Affiliations:** 1 Department of Oculoplasty and Ocular Oncology, Tilganga Institute of Ophthalmology, Kathmandu, Nepal; 2 Department of Research and Biostatistics, Tilganga Institute of Ophthalmology, Kathmandu, Nepal; Sanmenxia Central Hospital, Henan University of Science and Technilogy, CHINA

## Abstract

**Purpose:**

To evaluate the average motility of the implant and custom-made prosthesis (CMP) in various gazes following Myoconjunctival enucleation, the surgical duration, and the complications encountered at a tertiary eye hospital.

**Methods:**

This was a prospective, non-randomized interventional study. Recti muscles were sutured to the fornices before enucleation of the eyeball to minimize surgical time and the need for additional sutures. Thirty-five consecutive patients meeting the inclusion criteria were enrolled. Data on surgery time, complications, and implant/prosthesis motility were recorded and analyzed.

**Results:**

The mean surgical duration was 39.43 ± 4.97 minutes. Mean PMMA implant movement was 2.34 mm in elevation, 2.26 mm in depression, 2.74 mm in abduction, and 2.71 mm in adduction. Custom-made prosthesis (CMP) movement was 2.40 mm in elevation, 2.51 mm in depression, 2.74 mm in abduction, and 2.66 mm in adduction. Complications were minimal: one intraoperative superior rectus slippage, a few cases with early postoperative nausea, vomiting, and headache, all resolved within a week. At six weeks, one suture granuloma and one shallow inferior fornix were noted and managed. Patient satisfaction was high (88.57%), with a mean Likert score of 4.25 ± 0.65 (1 = Very unsatisfied to 5 = Very satisfied). No patient reported dissatisfaction.

**Conclusion:**

Myoconjunctival enucleation offers favorable implant and prosthesis motility, a relatively short surgical duration, and minimal complications. High patient satisfaction supports the myoconjunctival enucleation technique as a reliable and well-tolerated surgical option.

## Introduction

Enucleation, a procedure to remove the whole of the eyeball, is on the rise in many tertiary centers, primarily due to increased referrals for conditions such as intraocular tumors, phthisis bulbi, and end-stage glaucoma, among many others. In the traditional enucleation technique, the recti muscles are imbricated to one another, and a polymethyl methacrylate (PMMA) implant is used. This method is cost-effective, quick, and provides reasonable motility for patients [1,2]. PMMA implants are readily available and have been shown to be comparable to integrated implants in terms of efficacy and performance in a Cochrane intervention review [3]. Various integrated implants (e.g., hydroxyapatite, porous polyethylene, and composites) with recti muscles directly sutured to the implant or scleral coating over it are said to impart better motility and are commonly performed in developed countries [3].

The myoconjunctival enucleation technique involves suturing the recti muscles to the respective fornices and is aptly demonstrated in a video by Dr. Santosh G. Honavar [4,5]. This adds a few extra steps and sutures. This approach has been shown to provide superior motility to the prosthesis compared to traditional enucleation, significantly improving functional outcomes for anophthalmic patients in a study done by Shome et al. [1] Both aesthetic and functional motility are crucial for social acceptance and psychological well-being in such patients, contributing to enhanced patient confidence.

Despite its benefits, the Myoconjunctival technique is not widely practiced in Nepal. The use of five double-armed absorbable sutures and surgical time are limiting factors. There is a scarcity of published literature on its use in the region. We identified two randomized controlled trials from India, none from Nepal, and one cost analysis study from Canada [1,6,7]. Published studies from these countries have demonstrated that the technique provides excellent motility, low complication rates, and remains cost-effective. This study aimed to evaluate the effectiveness and patient satisfaction with the myoconjunctival technique within Nepal’s healthcare setting and advocate for its broader adoption.

## Materials and methods

Study design: This non-randomized prospective cohort study was conducted at Tilganga Institute of Ophthalmology, Kathmandu, from May 30, 2023, to December 2023, involving 35 eyes of the patients who underwent myoconjunctival enucleation for various indications. Patients aged over 18 years undergoing unilateral enucleation with a primary implant were included. Patients with enucleation without implant, conjunctival shrinkage, adnexal deformities, those scheduled for secondary implants, patients unwilling to participate, and those who could not attend scheduled follow-up were excluded from the study.

The study adhered to the tenets of the Declaration of Helsinki and was conducted in compliance with the guidelines of the Institutional Review Committee of Tilganga Institute of Ophthalmology. Ethical approval was obtained from the Nepal Health Research Council (NHRC) on May 10, 2023, with reference number 3054. Before enrolling the participants, the nature of the study and the responsibility of each participant were explained. Participants were also made aware of the freedom to withdraw from the study at any point in time. Informed written consent was obtained before inclusion into the study. Consent for the use of clinical data, photography, and videography of the evaluation process was obtained from each study participant. Based on the hospital records, in the previous year (2022), 35 cases underwent enucleation in the first 6 months. The study included 35 patients, representing all cases of myoconjunctival enucleation performed at our center over 1 year. Given the rarity of this procedure and the limited number of cases available, a formal sample size calculation was not performed before study initiation. Instead, we adopted a convenience sampling approach, with all eligible patients during the study period being included.

Surgical procedure: Under peribulbar anesthesia, after painting with 5% povidone-iodine and draping, conjunctival peritomy was done. Each rectus muscle was hooked, secured with a single half of 6−0 polyglactin suture (Vicryl, Ethicon, USA), detached from its insertion, and immediately attached to the corresponding fornix by making a loop. This step enabled fixation of all four recti to their respective fornices using a single suture, reducing both time and cost. The oblique muscles were cut, and any soft tissue attachments were released, and finally, the optic nerve was transected. Hemostasis was secured, and an appropriately sized polymethylmethacrylate (PMMA) implant was inserted in the intraconal space. The implant size was calculated by subtracting 2 mm from the axial length of the fellow normal eye of the patient [8]. According to the availability, the implant size was rounded to the nearest even number. The wound was closed in three layers: posterior tenon, anterior tenon, and conjunctiva. Posterior tenon and anterior tenon were sutured separately with multiple 4−0 polyglactin (Vicryl, Ethicon- USA) horizontal mattress sutures. The conjunctiva was sutured using 6−0 polyglactin in a continuous interlocking fashion. An appropriately sized conformer was placed, and temporary paracentral tarsorrhaphy was done. Any intraoperative complications and the surgery time were recorded. The enucleated specimen was sent for histopathological evaluation.

Postoperative care: All patients were given topical antibiotics and steroids in tapering frequency for six weeks after the surgery. Patients were followed up on postoperative day 1, day 7, 6 weeks, and 3 months. All the surgeries were done by one of the two experienced oculoplastic surgeons, Hom Bahadur Gurung (HBG) and Malita Amatya (MA). A custom-made prosthetic eye was fitted after 6 weeks of surgery for all cases. Complications on day 1, day 7, 6 weeks, and 3 months were recorded. The height, width, and thickness of the prosthesis were measured in millimeters, and volume was measured by the displacement method. Hertel’s exophthalmometry was done and recorded at 6 weeks.

**Fig 1:**
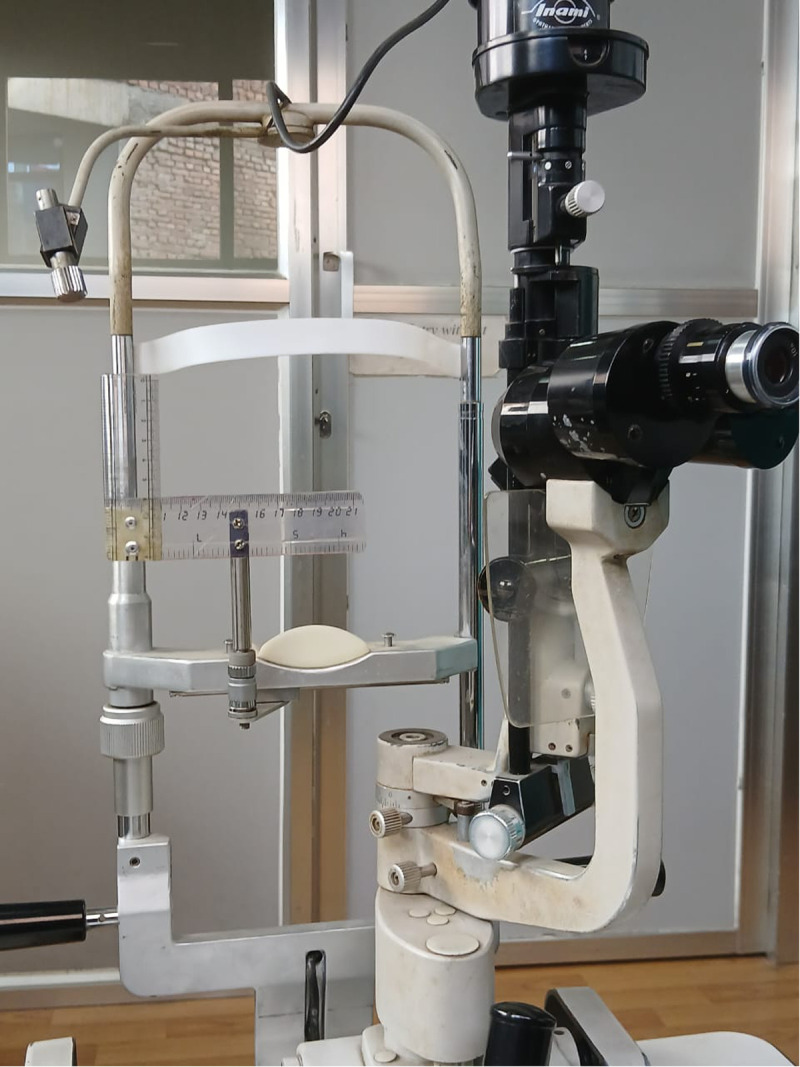
Customised slit lamp to measure implant and prosthesis motility.

Data collection: Implant and prosthesis movement measured 6 weeks after surgery was the primary outcome measure. A single observer measured the implant and prosthesis movement using a custom-made slit-lamp device for photographic documentation, described in a study by Raizada et al. [9] The device was fabricated with 2-mm scale rulers, having the dimensions of 15 cm and 5 cm, respectively. The larger ruler represented the x-axis, whereas the smaller ruler represented the y-axis, as shown in Fig 1. The larger horizontal ruler was fixed at the center, whereas the smaller vertical ruler could be moved along the x-axis.

Under topical anesthesia, with a wire speculum in place, the center of the horizontal palpebral fissure was marked on the conjunctiva using a nontoxic color marker. The movement of the conjunctival mark in adduction, abduction, elevation, and depression was photographed. The custom prosthesis was then placed in the socket. The prosthetic movement was measured in adduction, abduction, elevation, and depression with photographs and real-time video documentation. The range of implant and prosthesis movement in adduction, abduction, elevation, and depression from the reference point was measured. The measurements were manually estimated by an observer from video recordings of the implant and prosthetic motility. We used only one observer for consistency and logistical barriers, and we acknowledge the potential for observer bias.

Patients were asked, “Are the appearance and movement of the prosthesis satisfactory compared with your other eye?” at 6 weeks by the ocularist. Satisfaction was graded as “very satisfied”, “satisfied”, “not sure”, “dissatisfied”, and “very dissatisfied”, which corresponded to 5−1 on a Likert scale [10].

Data Analysis: Given the non-randomized, single-arm design and small sample size of the study, descriptive statistics were primarily employed. Implant and prosthesis motility measurements were summarized using means and standard deviations, while patient satisfaction scores were reported as frequencies and percentages. Due to limited statistical power, correlation and subgroup analyses were not conducted. Data cleaning was performed using Microsoft Excel, and statistical analysis was conducted using SPSS software. Where applicable, paired t-tests were used to compare means for statistical significance. A p-value < 0.05 was considered statistically significant.

## Results

Demographics: Table 1 summarizes the baseline demographic and clinical characteristics of the 35 patients undergoing Myoconjunctival enucleation with PMMA implant surgery. The cohort had a mean age of 31.83 ± 14.47 years (range: 18–70), with a nearly equal gender distribution (54.30% male, 45.70% female). 14 patients (40%) underwent right eye surgery, and 21 (60%) the left eye surgery. Axial lengths of the reference eye (non-operated eye) varied widely (20 to 30.11 mm, mean 22.90 ± 1.53 mm), reflecting diverse preoperative pathologies. The primary indications for enucleation were phthisis bulbi (n = 30, 85.7%), followed by staphyloma (n = 2, 5.7%), endophthalmitis (n = 1, 2.9%), panophthalmitis (n = 1, 2.9%), and painful blind eye secondary to corneal perforation (n = 1, 2.9%).

**Table 1:** Baseline demographic and clinical characteristics of patients undergoing orbital implant surgery.

Characteristic	Total(N = 35)
Age, years	
Mean ± SD	31.83 ± 14.47
Range	18-70
Sex, n (%)	
Male	19 (54.30)
Female	16 (45.70)
Operated eye, n (%)	
Right (OD)	14(40)
Left (OS)	21(60)
Axial length, mm	
Mean ± SD	22.90 ± 1.53
Range	20.0-30.11
Primary Diagnosis	
Phthisis bulbi	30(85.71%)
Other Diagnosis*	5(14.29%)

*Other diagnoses include endophthalmitis, panophthalmitis, staphyloma, and perforated corneal ulcer in a blind, painful eye with no visual prognosis.

**Fig 2:**
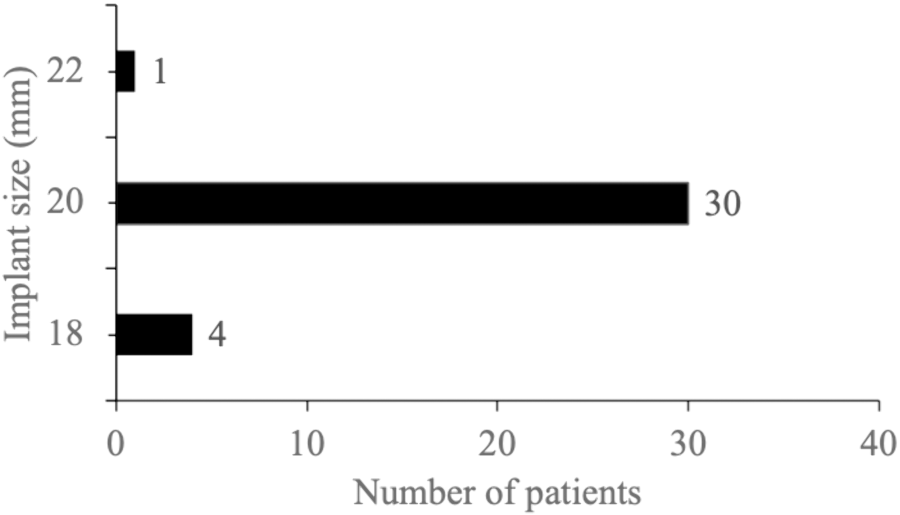
Frequency of patients with different implant sizes (N = 35).

Implant Size: The mean implant size was 19.83 ± 0.75 mm (range: 18−22 mm), consistent with the standard sizing formula (axial length – 2 mm) as described by Kaltreider and Lucarelli (Fig 2) [8].

Surgical time: The mean surgical duration was 39.43 minutes (SD = 4.97; range: 30–50 minutes). Most procedures (74%) were completed within 35–45 minutes, with a median duration of 40 minutes.

Implant and prosthesis motility: Ocular motility measurements demonstrated comparable movement between the implant and prosthesis across all directions (Table 2). Mean motility measurements ranged from 2.26–2.74 mm for implants and 2.40–2.74 mm for prosthesis across all directions. The range of implant motility was 1.0–4.0 mm in elevation and abduction, 1.0–3.0 mm in depression, and 2.0–4.0 mm in adduction. The range of prosthesis motility was 1.0–4.0 mm in depression, abduction, and adduction, and 1.0–3.0 mm in elevation. Functional motility ranges were preserved in all cases (1.0–4.0 mm). Paired t-tests revealed no statistically significant differences between implant and prosthesis motility in elevation, abduction, or adduction. Depression showed a small but statistically significant difference.

**Table 2:** Comparative motility between implant and prosthesis at 6 weeks (N = 35).

Movement	Implant (mm) (Mean ± SD)	Prosthesis (mm) (Mean ± SD)	Mean Difference (95% CI)	p-value
Elevation	2.34 ± 0.69	2.40 ± 0.65	−0.06 (−0.12, 0.24)	0.51
Depression	2.26 ± 0.56	2.51 ± 0.70	−0.25 (−0.38, −0.12)	0.03
Abduction	2.74 ± 0.66	2.74 ± 0.70	0.00 (−0.15, 0.15)	1.00
Adduction	2.71 ± 0.57	2.66 ± 0.87	0.05 (−0.10, 0.20)	0.47

Data presented as mean ± standard deviation. p < 0.05 is considered statistically significant.

Complications: Various complications observed during and after Myoconjunctival enucleation are depicted in Table 3. The slipped superior rectus was traced back and resutured to the fornix. Nausea, vomiting, and headache were the most common complications on the first postoperative day and were managed conservatively by oral/intravenous antiemetics and nonsteroidal anti-inflammatory drugs. One patient had a suture granuloma at 6 weeks’ follow-up and was excised under local anesthesia. Another had a shallow inferior fornix and was managed by a fornix deepening suture.

**Table 3:** Complications following Myoconjunctival enucleation and PMMA implant (n = 35).

Time Period	Complication	Cases (n)	Management approach
Intraoperative	Slipped superior rectus	1	Immediate reattachment to fornix
Postoperative Day 1	Nausea	9	Oral antiemetics
	Vomiting	6	Oral/IV antiemetics
	Headache	7	NSAIDs
Postoperative week 6	Suture Granuloma	1	Excision under local anesthesia
	Shallow inferior fornix	1	Fornix deepening suture

Satisfaction outcomes: At the 6-week postoperative evaluation, 88.57% of patients (31/35) reported being either satisfied (51.43%, n = 18) or very satisfied (37.14%, n = 13) with their prosthetic appearance and motility when compared to their unaffected eye, yielding a mean satisfaction score of 4.25 ± 0.65 (range: 3–5) on a 5-point Likert scale. Only four patients (11.43%) expressed neutral satisfaction (score = 3). Further exploration of reasons for neutral responses was not conducted, but two of them had reduced motility, and one had grade 1 socket contracture. No patients reported active dissatisfaction (scores 1–2).

## Discussion

Enucleation may be the oldest operation in ophthalmology, and has been mentioned as early as 2600 BC [11]. Enucleation is the first step to a long journey, and a surgeon can make a difference with the enucleation technique and the choice of implant. Replacing adequate orbital volume, maximizing prosthetic movement, comfort, and aesthetic appearance should be the ultimate goal. Management of an anophthalmic socket involves using the proper surgical technique, choosing the right implant (such as non-integrated or integrated), and ultimately the prosthesis. Orbital implants began when Mules implanted a glass sphere after an evisceration in 1885 [11]. Various implant materials, shapes, and sizes have been developed since then. The orbital implant may or may not be wrapped. The rectus muscles can be imbricated (routine enucleation) to each other, attached to fornices (myoconjunctival technique), or attached to wrapped material or an implant itself.

Direct suturing of extraocular muscles to the fornices allows movement of the muscles to be transmitted directly to the fornices. Coston is credited as the first person to suture muscles to the fornix [12]. Since the pulling effect on the fornices is the most important determinant of movement with the spherical implant, the motility is maximized. Nunery et al. described the technique of myoconjunctival enucleation in 25 patients [4]. The technique uses relatively lower-cost spherical implants and has been shown to impart good ocular motility, so it is frequently used in different parts of the world [1]. In an international survey on retinoblastoma, 19% of surgeons preferred myoconjunctival enucleation [13].

The implant and prosthesis motility measurements were comparable in all gazes in our study. The mean motility measurements ranged from 2.26–2.74 mm for implants and 2.40–2.74 mm for prosthesis in different gazes. There was no statistically significant difference between the two in elevation, abduction, or adduction. However, depression showed a small (0.25 mm) but statistically significant difference (p = 0.03). This difference is below the 0.5 mm threshold considered to be clinically meaningful in prosthetic motility assessment [8,14,15]. . This variation likely reflects measurement variability rather than functional limitation. We identified only three studies studying myoconjunctival enucleation outcomes. The first was a nonrandomized comparative study between routine enucleation and Myoconjunctival enucleation by Yadava U et al. [6] They had 15 cases in both groups, and they used Nunery and Chen‘s modification of myoconjunctival enucleation, where they sutured the extraocular muscles to the sclera, a wrapped silicone implant, and to the respective fornices. They did not mention standard deviation in their results, so statistical analysis could not be done, but the means of implant motility were higher than in our study in all four gazes. The method of measurement was also different from our study. We followed a similar surgical and measurement technique to that in the study by Debraj Shome et al. [1,9] It was a randomized controlled trial comparing implant and prosthetic motility among three groups: traditional enucleation with PMMA, myoconjunctival enucleation with PMMA, and porous polyethylene implant. Compared to the myoconjunctival PMMA group in Shome et al., our study demonstrated significantly lower motility in both implant and prosthesis measurements across all gazes (p < 0.01). Our result was also skewed due to a case of corneal perforation following a corneal ulcer with conjunctival congestion, which had very limited motility at 6 weeks. All other cases were non-inflamed conjunctivae. Active conjunctival inflammation may have contributed to the decreased motility of the implant and prosthesis. The results may also have been influenced by surgeon experience, potential variation in surgical procedure, differences in patient characteristics, and quality of custom-made prosthesis. However, both the implant and prosthesis measurements in our study were better than the traditional PMMA group in the Shome et al. study (p < 0.01 in adduction, elevation, and depression). Hence, it suggests that myoconjunctival enucleation is better than the traditional enucleation technique. The third study by Asari et al. was a prospective analytical study of myoconjunctival enucleation with 35 patients, and only implant motility was measured with a standard millimeter ruler [2]. The mean implant motility was similar to our study, but since the standard deviation was not given, statistical comparison could not be performed.

Our surgeries took around 4.5 minutes longer (39.3 minutes versus 34.8 minutes, p = 0.005) than the Shome et al study. This may be due to surgeon experience, protocol differences, or variation in surgical technique.

Regarding complications, intraoperative slippage of the superior rectus muscle occurred in one case but was successfully retrieved and resutured. Two cases required canthotomy, and one case required cantholysis as an added procedure. We do not routinely do canthotomy and cantholysis, but the eyeball was large in these cases, and canthotomy and cantholysis gave more space for enucleation. Nausea, vomiting, and headache were common on the first postoperative day, and two patients required intravenous antiemetics; all of these symptoms resolved by one week. One patient had a suture granuloma at six weeks and was excised under local anesthesia, while one had shallow inferior fornix, and a fornix deepening suture was applied. There was no implant-related complication in any patient. Our implant was central in all cases until the last follow-up, like all other studies. In the Myoconjunctival technique, the implant is inserted deep in the intraconal space and covered by layers of posterior tenon, anterior tenon, and conjunctiva. Hence, it is stable and unlikely to be displaced or extruded. Shome et al. reported no complications in their myoconjunctival enucleation group [1]. In contrast, Yadava et al. observed deep superior sulcus and mild ptosis in all cases, which was not observed in our cohort [6]. In another study analyzing complications between myoconjunctival and conventional techniques for retinoblastoma, conjunctivitis, implant extrusion or exposure, and cellulitis were seen in 16% of myoconjunctival enucleation [7]. One patient with pre-existing phthisis bulbi and ptosis underwent frontalis sling surgery after 3 months.

A custom-made prosthesis was made for all patients. The length, width, and thickness of all prostheses were measured. Hertel’s ophthalmometry was done at 6 weeks. Only 4 patients had a difference of 2 mm between the normal eye and prosthetic eye. One had staphyloma in both eyes, so she had the highest difference of 6 mm. We used a 20 mm implant size in about 85.71% which is similar to the study by Song et al [10].

13 patients (37.14%) said they were very satisfied and 18 patients (51.42%) said they were satisfied, and 4 (11.43%) were unsure. The only study that surveyed satisfaction in anophthalmic patients wearing ocular prosthesis had a 71.8% overall rate of satisfaction with ocular prosthesis [10]. However, we did not do an in-depth analysis of why they were not satisfied or which factors were correlated with patient satisfaction. Among the unsure patients, two had reduced motility, and one had a contracted socket (shallow inferior fornix). The patient satisfaction score generally represents the patient’s course of treatment and satisfaction with the surgical procedure and prosthesis. It may not represent the true satisfaction of the surgical procedure alone. Notably, no patients reported active dissatisfaction (scores 1–2), underscoring the overall success of the surgical and prosthetic rehabilitation process.

## Limitations

It is a single-arm study. The superiority of the technique over other techniques cannot be established. Blinding of observers was not performed and may lead to bias. We had a single observer, which might have caused observer bias. Satisfaction was measured simplistically due to resource constraints; future work will benefit from the use of multidimensional tools. The study is limited by a relatively small sample size, which may affect the generalizability and statistical power of subgroup or correlation analyses. However, this reflects the real-world clinical volume of myoconjunctival enucleation procedures over a year, and to our knowledge, represents one of the few focused prospective data sets from the region.

## Conclusion

Myoconjunctival enucleation with PMMA implant is a good and cost-effective option for providing functional motility to the prosthesis with minimal complications. It provides better implant and prosthesis motility than traditional enucleation and PMMA implant. With minimal modification in the suturing technique, the cost of sutures and overall surgical cost can be cut down. The surgical procedure, coupled with a custom-made prosthesis, resulted in high satisfaction among patients.

## Recommendation

A randomized controlled trial between traditional enucleation (the most commonly performed enucleation in Nepal) and myoconjunctival enucleation should be done. Though RCTs have been done in India, myoconjunctival enucleation is not established among oculoplastic surgeons in the country.

## Supporting information

S1 Dataset Raw anonymized dataset used for statistical analysis in the study. (XLSX)
